# Spatial beam reshaping and large-band nonlinear conversion in rectangular-core phosphate glass fibers

**DOI:** 10.1007/s12200-022-00007-6

**Published:** 2022-03-29

**Authors:** Clément Strutynski, Vincent Couderc, Tigran Mansuryan, Giorgio Santarelli, Philippe Thomas, Sylvain Danto, Thierry Cardinal

**Affiliations:** 1 Institute of Chemistry of the Condensed Matter of Bordeaux (ICMCB), Chemistry Department, 33608 Pessac, France; 2 UMR CNRS 7252, Université de Limoges, XLIM, 87060 Limoges, France; 3 LP2N, Institut d’Optique Graduate School– CNRS–University of Bordeaux, 33400 Talence, France

**Keywords:** Phosphate glasses, Neodymium, Optical fibers, Self-focusing, Super-continuum

## Abstract

Here we present the ability of Nd^3+^-doped zinc-phosphate glasses to be shaped into rectangular core fibers. At first, the physico-chemical properties of the developed P_2_O_5_-based materials are investigated for different concentrations of neodymium oxide and core and cladding glass compositions are selected for further fiber development. A modified stack-and-draw technique is used to produce multimode large rectangular-core optical fibers. Self-guided nonlinear effects acting as spatial beam reshaping processes occurring in these newly-developed photonic structures lead to the generation of spectral broadenings in the visible and near-infrared spectral domains.

**Graphic Abstract:**

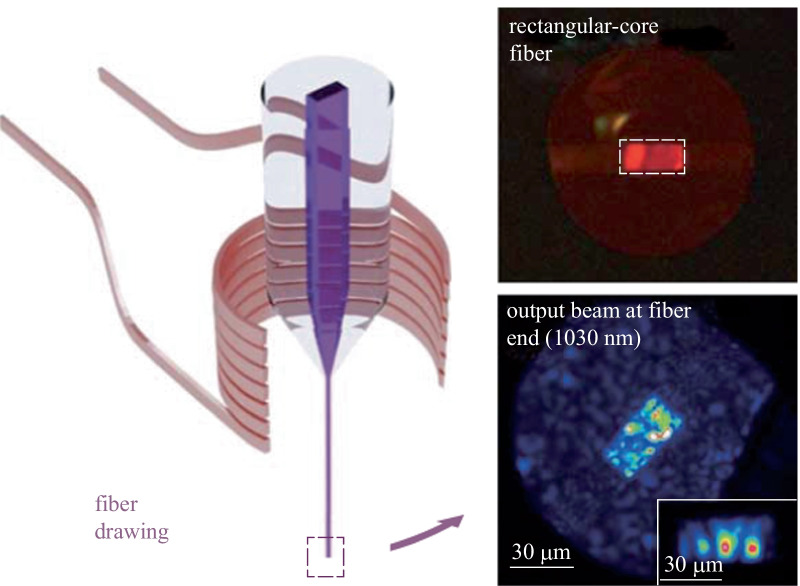

## Introduction

Multimode graded index optical fibers have recently received strong interest because of their ability to transform a multimode propagation into a quasi-single mode one. This effect, called spatial self-cleaning, is based on four-wave mixing (FWM) process, which is phase matched by a longitudinal grating formed by the interplay between a spatial self-imaging and the Kerr effect [1]. Thus, high peak power can be guided in multimode fibers without significant spatial spreading of the energy in a speckled beam. Combined with special active rare-earth doped gain media, the exploitation of such dynamics has proven promising for the power scaling of all-fibered laser sources [2]. Alternatively, nonlinear spatial cleaning of multimode beams can advantageously provide strong power density inside the waveguiding structure and lead to supercontinuum (SC) generation [3, 4]. Broadband generation by using nonlinear conversions was first introduced in 1970 by Alfano and Shapiro in bulk glass [5]. Later, the advent of microstructured optical fibers, in the 1990s [6], attracted new interest, guided by the design of the curve dispersion of nonlinear fibers. Thus, the control of nonlinear processes at the origin of the supercontinuum generation has been possible. Broadband lasers became the usual sources for many applications, such as nonlinear fluorescence imaging and multispectral Lidar. Pulsed laser sources ranging from femtosecond [5] to nanosecond [7] have been used without forgetting the continuous wave (CW) regime [8]. Detailed explanations of nonlinear processes involved in supercontinuum generation can be found in the paper published by Dudley et al. in 2006 [9]. Up to now, graded index optical fibers have been the main photonic structures which can support spatial self-cleaning processes. However, it is interesting to underline that non-cylindrical fiber geometries can also be considered to transfer power, propagating on the fundamental mode toward higher-order modes (HOMs), by exploiting nonlinear dynamics [10]. Besides, linear and cubic nonlinear imaging effects were experimentally investigated in slab waveguides [11]. Building on this, it appears that the development of rectangular fibers provides very interesting features for nonlinear spatial reshaping effects and for frequency conversion [12, 13]. These unique structures were initially investigated on silicate glasses for their better thermal management and pump absorption efficiency. They can, however, be designed from other materials exhibiting more suitable assets for laser applications, such as phosphate glasses. P_2_O_5_-based materials provide high solubility of rare-earth ions, up to several 10^20^ ions/cm^3^, good chemical durability, excellent optical properties and fiber-shaping ability [14, 15]. Previous studies have indicated that they overcome several silica-related limitations (photo darkening, Brillouin scattering, etc.) in the domain of fiber [16–20]; shorter [21–23] or high power [24, 25] fibered laser configurations are already available with these glasses. As an illustration, Nd^3+^-doped metaphosphate has been extensively investigated for the development of high energy and high-peak-power laser applications such as the multi-kilojoule, multi-terawatt lasers for fusion energy research ignition facilities [26]. Recently, a single-frequency laser operation in the 900 nm wavelength range with an output power of 13.5 mW has been demonstrated using an Nd^3+^-doped phosphate fiber as the gain medium [27].

In this paper, we present an overview of the fabrication process and characterization of Nd-doped zinc-phosphate rectangular-core step-index fibers. First, the effect of Nd_2_O_3_ addition on the physicochemical properties of the glass 55P_2_O_5_–30ZnO–10K_2_O–5Al_2_O_3_ is studied. Thermal and optical properties show linear evolution with respect to the Nd^3+^ concentration, allowing fine control of the glass transition temperature and of the refractive index. Core and cladding glasses were selected based on these investigations and a rectangular-core multimode optical fiber was manufactured using a modified stack-and-draw technique recently developed by our group [28]. Thereafter, femtosecond laser pumping was employed to demonstrate the generation of a large-band spectral broadening in these newly-developed fibers thanks to self-guided nonlinear effects.

## Experimental procedure

### Glass preparation

Phosphates glasses of composition (55P_2_O_5_–30ZnO–10K_2_O–5Al_2_O_3_)_100−*x*_ + *x*Nd_2_O_3_ (*x* = 0.25, 0.5, 1.0, 1.5, 2.0 and 4.0) (in mol. %) were studied. Here, Nd_2_O_3_ was employed to finely tune the specific thermal and optical properties of the glass in view of core and clad fiber fabrication. The glasses were prepared by the standard melt-quenching technique. High purity (99.999%) precursors (H_3_PO_4_, ZnO, K_2_CO_3_, Al_2_O_3_, and Nd_2_O_3_) were ground and placed together in a platinum crucible and melted at 1100 °C for several hours. Stirring and grinding operations were performed during the synthesis to improve glass homogeneity. 8 mm diameter and ~ 6 cm long mono-material glass rods were fabricated from ~ 15 g batches. After quenching, the samples were annealed at 10 °C below their glass transition temperature for 8 h to reduce internal stress. Glass slabs of a few millimeter-thick each were cut from the cylindrical preforms and subsequently polished for optical characterizations.

The glass preforms were drawn under an Ar flow (2 L/min) using a dedicated 3 m high draw tower at ~ 560 °C–600 °C. First the macroscopic arrangement was heated up to its softening temperature at 10 °C/min to initiate the elongation process. After that, the preform was slowly fed into the furnace while the drawing parameters were continuously monitored to produce a fiber with a constant outer diameter and thus ensuring the uniformity of the profile along the fiber length. Further experimental details are provided below regarding the assembling of the preforms and their drawing into neodymium-doped multimode fibers.

### Thermal and physical properties

The glass transition temperature (*T*_g_) and crystallization temperature (*T*_c_) were determined through differential scanning calorimetry (DSC) measurements with precision of ± 3 °C. DSC curves of ~ 60 mg glass samples were registered between 20 °C and 700 °C with a heating rate of 10 °C/min under ambient air. *T*_g_ was taken at the inflection point of the endotherm, as obtained by taking the first derivative of the DSC curve. *T*_c_ is defined as the onset of the exothermic peak.

The density of the bulk glass materials was determined through Archimedes’ method in Diethyl phthalate. The precision was better than ± 0.02 g/cm^3^.

### Optical properties

Refractive indices were measured at 656 nm using Abbe refractometer (Atago). Optical loss measurements were carried out using the cutback method on fiber samples of several meters each. A 1310 nm laser source, with power of a few mW, was coupled into the phosphate fiber by the means of a silica objective (20 × and 0.32 NA). The output power was then measured for different sample lengths using a power-meter with µW sensitivity.

### Nonlinear experiments configuration

SC generation was obtained by pumping the multimode rectangular-core phosphate fiber with a 1030 nm laser source delivering 250 fs pulses at a repetition rate of 300 kHz with a maximum average power of 2 W. The laser beam was coupled to the phosphate waveguides using a convergent lens (*f* = 175 mm) (coupling efficiency estimated to ~ 50%). The generated spectral broadenings were collected by directly aligning the output of the phosphate fiber samples with a multimode silica collection fiber. The signal was then analyzed with an optical spectrum analyzer operating in the 350–1750 nm range.

## Results and discussion

### Material development

Here, we chose the P_2_O_5_–ZnO–K_2_O–Al_2_O_3_ system for multimode step-index fiber development. Often phosphate vitreous materials suffer from poor mechanical properties and low chemical resistance, especially against exposure to humidity. However, when tailored with the appropriated addition of oxide compounds, phosphate glasses have proven to form vitreous matrices suitable for optical fiber development, with good chemical durability and excellent shaping ability [14, 15]. Presence of the network intermediate Al_2_O_3_ increases the glass stability [29] and reduces rare-earth ions clustering [30] while ZnO helps preventing devitrification [31, 32]. It is worth noting that the alkali metal oxide K_2_O was added, in the work reported in this paper, to lower the glass characteristic temperatures (glass transition and melting temperatures) for better rare earth oxide solubility and easier performance of shaping.

Glasses of composition (55P_2_O_5_–30ZnO–10K_2_O–5Al_2_O_3_)_100−*x*_ + *x*Nd_2_O_3_ (*x* = 0.25 to 4.0) were synthesized here, and their main properties are summarized in Table 1.

**Table 1:** Physicochemical properties of the glasses in the system (55P_2_O_5_–30ZnO–10K_2_O–5Al_2_O_3_)_100−*x*_ + *x*Nd_2_O_3_ (*x* = 0.25, 0.5, 1.0, 1.5, 2.0 and 4.0) (in mol. %)

Nd_2_O_3_ doping		*T*_g_	*T*_c_	Δ*T*	Density	[Nd^3+^]	*n*_@656 nm_
/(mol. %)	/(wt. %)	/ ± 3 °C	/ ± 3 °C	/ ± 6 °C	/ ± 0.02(g·cm^−3^)	/(10^20^ ions·cm^−3^)	(± 0.001)
0	0	417	n.m.	> 283	2.80	0	1.528
0.25	0.72	420	n.m.	> 280	2.82	0.72	1.531
0.5	1.42	422	n.m.	> 278	2.85	1.45	1.532
1.0	2.82	430	n.m.	> 270	2.88	2.91	1.533
1.5	4.20	435	n.m.	> 265	2.89	4.34	1.535
2.0	5.54	438	555	117	2.90	5.75	1.536
4.0	10.70	456	590	134	2.95	11.30	1.540

**Fig. 1:**
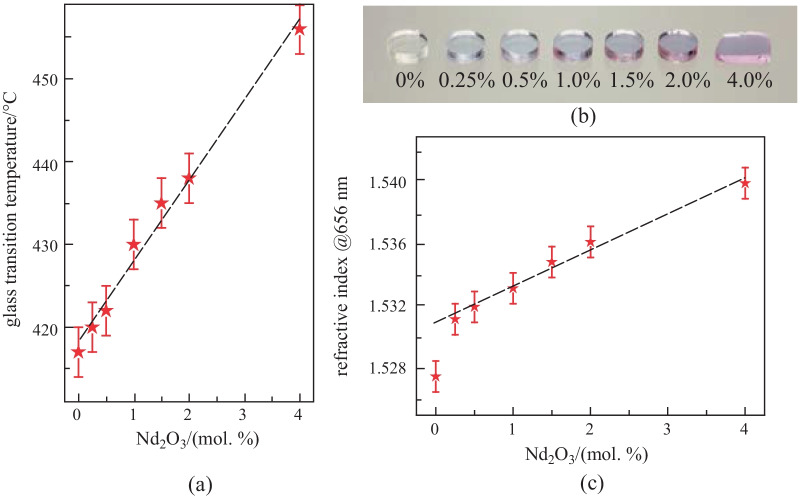
Evolution of **a** glass transition temperature, **b** glass samples, and **c** refractive index at 656 nm as function of Nd_2_O_3_ doping level (in mol. %)

Evolution of the glass transition temperature with respect to neodymium oxide content is plotted in Fig. 1a. It reveals that addition of Nd_2_O_3_ changed the thermal characteristics of the host zinc-phosphate glass. The glass transition temperature indeed gradually increased with higher neodymium oxide content. This indicates that neodymium ions brought greater rigidity and connectivity to the glassy network by increasing cross-linking in a similar fashion as is achieved by using aluminum oxide [29]. No crystallization temperature was visible at the 10 °C/min measurements rate for the different samples, except for the 2.0% and 4.0%, confirming the good stability of the host matrix. All glass samples exhibit *T*_c_−*T*_g_ differences greater than 100 °C and are therefore thermally suitable for fiber drawing.

A picture of the samples investigated here is shown in Fig. 1b. The gradual coloration of the glass is due to the multiple visible absorptions related to 4*f*^3^ transitions of neodymium [33, 34]. The samples showed good homogeneity and overall fine neodymium incorporation even for important doping levels. High solubility of rare-earth ions is one of the major assets of use of phosphate glasses. It ensured good pump conversion over short propagation distances and could further allow the development of extremely compact, short laser systems.

As expected, the glass density increased with the addition of Nd_2_O_3_, which possesses a high molar mass (336.48 g/mol) as compared to the calculated host matrix molar mass (117.01 g/mol). The refractive index followed a comparable linear trend as depicted in Fig. 1c. It increased from 1.528 for the undoped glass up to 1.540 for the sample containing the highest Nd_2_O_3_ proportion. Addition of Nd^3+^ ions led to the presence of more non-bridging oxygen atoms within the glass matrix, producing an increase of electron polarizability [35].

Evolution of the index is of great interest for step-index optical fiber manufacturing which needs fine control of refractive index difference between the core and the cladding materials. This is especially true for laser applications which require on one hand large cores to increase the available output power, but on the other hand low numerical aperture (NA) to keep the single- or quasi single-mode operation and guaranty satisfactory beam quality. Here, when considering the undoped and Nd-loaded glasses as respectively cladding and core materials, the Nd_2_O_3_ concentration could be set to target specific refractive index steps for the final fiber.

### Neodymium-doped multimode fiber fabrication

**Fig. 2:**
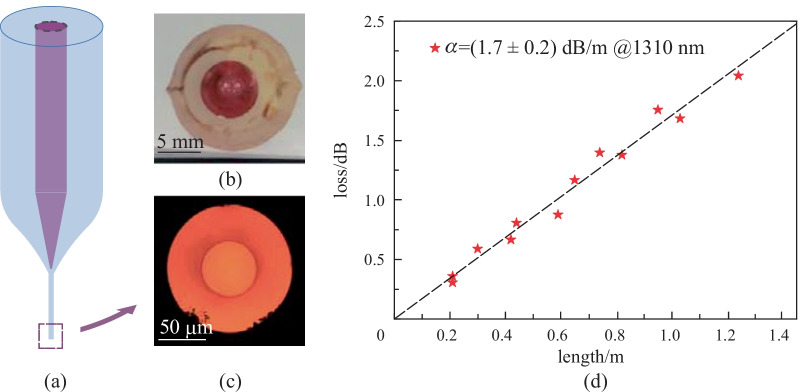
**a** Thermal drawing of a large-core cylindrical phosphate fiber. Cross-sectional view of **b** step-index glass preform and **c** neodymium-doped multimode fiber. **d** Optical loss of the MMF measured at 1310 nm

In a first step, a cylindrical multimode fiber (MMF) was fabricated by exploiting the vitreous materials developed in the previous section. A large-core preform was manufactured using the built-in casting method [36]. The undoped glass was chosen for the cladding material while the glass doped with 1.0 mol. % Nd_2_O_3_ was selected for the core. A cross-sectional view of a typical step-index rod is presented in Fig. 2b. The refractive index difference between the core and the cladding was (5 ± 2) × 10^−3^ (at 656 nm) and the glass transition temperature difference Δ*T*_g_ = (13 ± 6) °C, meaning the two glasses were thermally and optically compatible for fiber development. The preform was then stretched down in homothetic fashion to a multi-mode fiber for further characterizations. A cross-sectional view of the as-drawn MMF is presented in Fig. 2c. No index fluctuation (glass striae) was visible on the fiber microscope pictures taken in transmission mode, which attests good homogeneity of the core glass. Additionally, the structure possessed a good core-cladding interface without particular defects (bubbles, crystallites, etc.). Those observations were confirmed by the attenuation at 1310 nm which was found to be as low as 1.7 dB/m (see Fig. 2d). It is worth noting that this value was comparable to the attenuation measured on single-index cylindrical fibers made from the cladding or core glass (respectively 1.4 and 1.8 dB/m). The properties of this cylindrical MMF are summarized in Table 2.

**Table 2:** Properties of the P_2_O_5_-based multimode fibers (MMF) investigated in this section

	Nd_2_O_3_ core doping	Δ*T*_*g*_	Δ*n*_@656 nm_	Optical loss at 1310 nm/(dB⋅m^−1^)		
	/(mol. %)	(± 6)/°C	(± 0.002)	Cladding	Core	MMF
Cylindrical MMF	1	13	5ּ 10^−3^	1.4	1.8	1.7
Rectangular MMF	1.5	18	7ּ 10^−3^	1.4	1.9	14*

* Optical loss measured at 1030 nm

The development of multi-mode cylindrical waveguides allowed assessment of the ability of the phosphate glass to be shaped into fibers of optical quality. Regarding the direct preform-to-fiber fabrication of a rectangular-core fiber, we selected the glass doped with 1.5% neodymium oxide as the core material because of its high Nd_2_O_3_ content combined with its excellent thermal stability. The undoped glass was chosen as the cladding material. The refractive index difference between the core and the cladding was (7 ± 2) × 10^−3^ (at 656 nm), producing a fiber with a numerical aperture of 0.15. The core and the cladding both exhibited *T*_c_*–T*_g_ differences greater than 100 °C and were therefore thermally suitable for fiber drawing. Additionally, they showed a moderate glass transition temperature difference Δ*T*_g_ = (18 ± 6) °C, meaning they were thermally compatible for co-drawing. Again, optical loss measurements were carried out on single-index cylindrical fibers to verify the optical quality of the core material doped with 1.5 mol. % Nd_2_O_3_ (Table 2). A moderate attenuation of 1.9 dB/m was measured at 1310 nm, which was sufficient for the nonlinear experiments described below, involving short fiber segments.

**Fig. 3:**
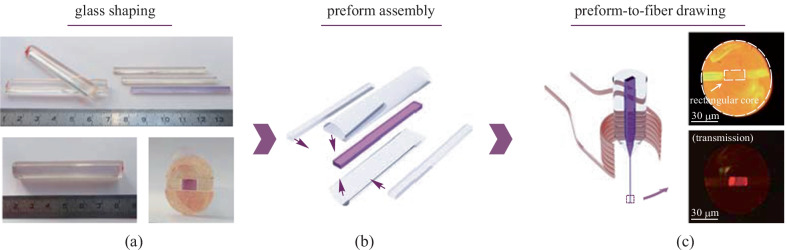
**a** Pictures of the different phosphate glass parts used for the preform design. **b** Description of the preform assembly and **c** thermal drawing of rectangular-core phosphate fibers

Then, a glass preform possessing a rectangular core was fabricated using a modified stack-and-draw technique as outlined in Fig. 3 [28].

The different parts of the preform were cut out from glass rods and plates fabricated with the melt-quenching technique. Two half-cylinders and three rectangular canes were used here (see Fig. 3a). Those parts were then carefully polished to produce high quality surfaces and put together to form the preform. The assembly was then annealed for two hours at *T*_g_ + 50 °C while a moderate pressure (few kPa) was applied to the glass pieces by means of ceramic weights. This step allowed for the bonding of the different glass parts and ensured good and permanent contact between them. This procedure, also known as fusion bonding, is widely used with silicate glasses in microfluidics for micro-chip assembly or for silicon wafer assembly [37]. The glass pieces bond together as a result of a reaction between the Si–OH groups existing at the surface of the slabs. Upon heating, H_2_O is released while Si–O–Si entities form, bonding the glasses together [38]. In the present case, P-OH or M-OH (M for cations) present at the surface of the phosphate glasses reacted when heated up, releasing molecular water and forming P-O-P or P-O-M bonds, bridging the glass pieces together. This procedure is of great importance for fiber drawing as it gives the needed cohesion to the preform built from the different glass pieces. After that, the preform was thermally scaled down in a homothetic fashion to produce tens-of-meter long rectangular-core optical fibers. Light guiding inside the non-cylindrical core of the produced wave-guide was assessed as shown in Fig. 3b.

### Supercontinuum generation

**Fig. 4:**
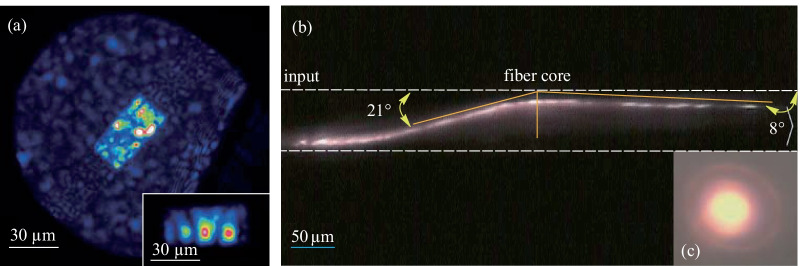
**a** Image of the output beam at the fiber end. The guided zone is estimated to 30/60 µm; Inset: example of excitation of few modes by controlling the input coupling. **b** Nonlinear propagation of the incident beam inside the core fiber and leading to nonlinear refraction evolution (Input peak power: 1.6 MW). **c** Far field image of the output visible beam for peak power of 2 MW

A fiber with a rectangular-core of 30 µm in thickness to 60 µm in width was fabricated following the method described previously. The number of supported modes at 1030 nm was estimated at ~ 95 in such a structure. Thus, the purpose of the first experiment was to observe the output beam shape at the fiber end after the coupling of a 1030 nm Gaussian beam with 25 µm diameter (see Fig. 4a). In these conditions, the input coupling could select different groups of modes in order to provide different output beam patterns. In this case, the selection of a low number of modes was possible, leading to the reduction of the output speckle (see inset of Fig. 4a). The optical loss in the fiber was measured by using the cut-back technique and was estimated to be close to 14 dB/m, which was well above the values determined on single-index fibers or on the cylindrical MFF (see Table 2). That high coefficient most probably originated from poor core-cladding interfaces where diffusion centers (glass stria, interface imperfections, bubbles) could form during the thermal drawing. In particular, when comparing this value to the 1.7 dB/m attenuation measured on the cylindrical MMF, it appeared that the high optical loss measured on the rectangular-core fiber did not derive from poor material quality but rather from the waveguide geometry and the preform manufacturing process. Further improvement, including optimization of the fabrication protocol and modeling to optimize the geometry of the rectangular core must be considered in order to lower the loss value.

**Fig. 5:**
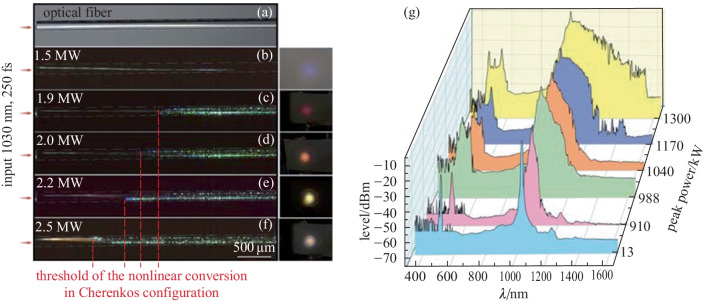
Longitudinal view of the rectangular fiber during nonlinear conversion process versus the input peak power. Fiber without pumping **a**, fiber pumped at 1030 nm, 250 fs versus input peak power **b** −** f**. Insets on the right side of the figure correspond to the output beam pattern observed in the visible domain after 45 cm of propagation. **g** Spectrum recorded at the fiber end versus the input peak power

In the second step we investigated the supercontinuum generation by using femtosecond pulses. To counterbalance the dispersion and the group velocity difference between modes, the peak power was significantly increased in order to reach the self-focusing propagation regime (Figs. 4 and 5). In these conditions, the first nonlinear signature appeared in the spatial domain with nonlinear self-focusing which significantly changed the spatial beam pattern and drastically improved the local power density inside the fiber. The nonlinear multimode filament was perfectly guided in the fiber core and underwent spatial reorientation because of the nonlinear interactions between transverse modes supporting different energies. Thus, the linear refraction process, well known in the optical fiber, was no longer conserved and nonlinear reflection could be observed. Figure 4b shows an example of that process with a quasi-total reflection at the core cladding interface, changing an incident angle of 21° into an output one close to only 8°. This atypical process had a strong influence on the beam propagation and also on the frequency conversion. The nonlinear propagation regime was observed on the side of the optical fiber because of non-collinear frequency conversion obtained on leaky modes and by means of a Cerenkov phase-matching process. The signature of that non colinear conversion was clearly visible on the far field image of the converted output beam with a thin ring surrounding the main central spot (Fig. 4c). However, because of the high numerical aperture of the fiber, a large part of the converted beam was collected on the central beam, constituted of several transverse modes. After a given propagation length, the impact of the nonlinearity reached a critical level and broke the initial white filament (see Fig. 5a–f). Thus, a nonlinear frequency conversion could be obtained in the infrared domain by using self-phase modulation and the Raman effect.

A supercontinuum up to 1550 nm was observed for a peak power close to 1.9 MW (Fig. 5c). The conversion toward the visible region was first obtained, in part, by second harmonic generation (SHG) at 515 nm and was followed by four-wave mixing processes which enlarged the visible spectrum (Fig. 5g). The phase matching process regarding the SHG was obtained thanks to non colinear phase matching configuration [39, 40]. Phase matching of additional v was mainly obtained by modal mixing. The higher the input power, the earlier the nonlinear conversion appeared in the optical fiber (see Fig. 5b–f).

## Conclusions

A detailed study concerning the development of zinc-phosphate glasses doped with different Nd_2_O_3_ concentrations was carried out. Linear evolution of the glass properties (glass transition temperature and refractive index) with respect to Neodymium oxide concentration was observed. Then, thanks to the good fiber drawing ability of the developed glasses, multimode large rectangular-core fibers were produced by a modified stack-and-draw method. Self-guided nonlinear effects, acting as a spatial beam reshaping which concentrated the input energy, facilitated large-band frequency conversion in the visible and infrared domains in this new waveguide structures. An additional spatial routing of self-focused filament due to interplay between multimode propagation and nonlinearity was also observed. Further work will be devoted to determine whether the nonlinear dynamics exploited here are compatible with gain in the fiber.
